# Supplementary material for: Transmission-blocking activities of artesunate, chloroquine, and methylene blue on Plasmodium vivax gametocytes
Source: Antimicrob Agents Chemother. 2024 Jul 26;68(9):e00853-24. doi: 10.1128/aac.00853-24 (PMC11382624; doi:10.1128/aac.00853-24)
Supplement: Supplemental material — Table S1; Figures S1 to S6. [file aac.00853-24-s0001.docx]

Table S1. Summary of mosquito assay runs.

| Drug | No. blood samples | No. mosquito batches | | | No. dissected guts | | | **No. dissected pairs of salivary glands** | | |
| --- | --- | --- | --- | --- | --- | --- | --- | --- | --- | --- |
|  |  | **Base.** | **Cont.** | **Treat.** | **Base.** | **Cont.** | **Treat.** | **Base.** | **Cont.** | **Treat.** |
| Artesunate | 9 | 9 | 36 | 36 | 450 | 1797 | 1798 | 90 | 360 | 360 |
| Chloroquine | 21 | 21 | 84 | 84 | 1008 | 4026 | 4036 | 263 | 1228 | 1177 |
| Methylene blue | 8 | 8 | 32 | 32 | 404 | 1592 | 1599 | 80 | 320 | 320 |

Abbreviation: No; number, base; baseline, cont; control, treat; treated.


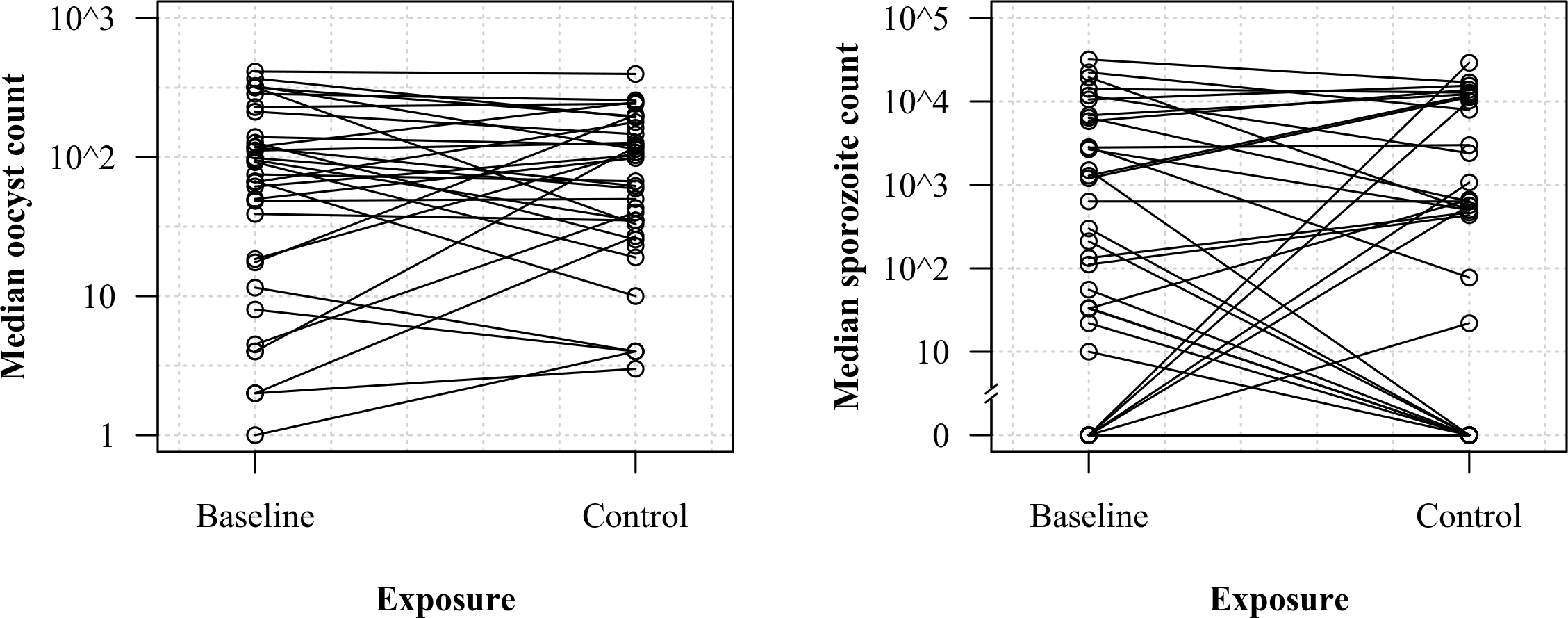
**Figure S1.**

**Evolution of sample infectiousness to mosquitos during incubation ex vivo.** Variations in the median oocyst (left panel) and median sporozoite count (right panel) between baseline (i.e., on the day of sample collection, before incubation) and control (i.e., after 24 hours of incubation without drug) mosquito batches. Values of experimental replicates were collated by assay run.


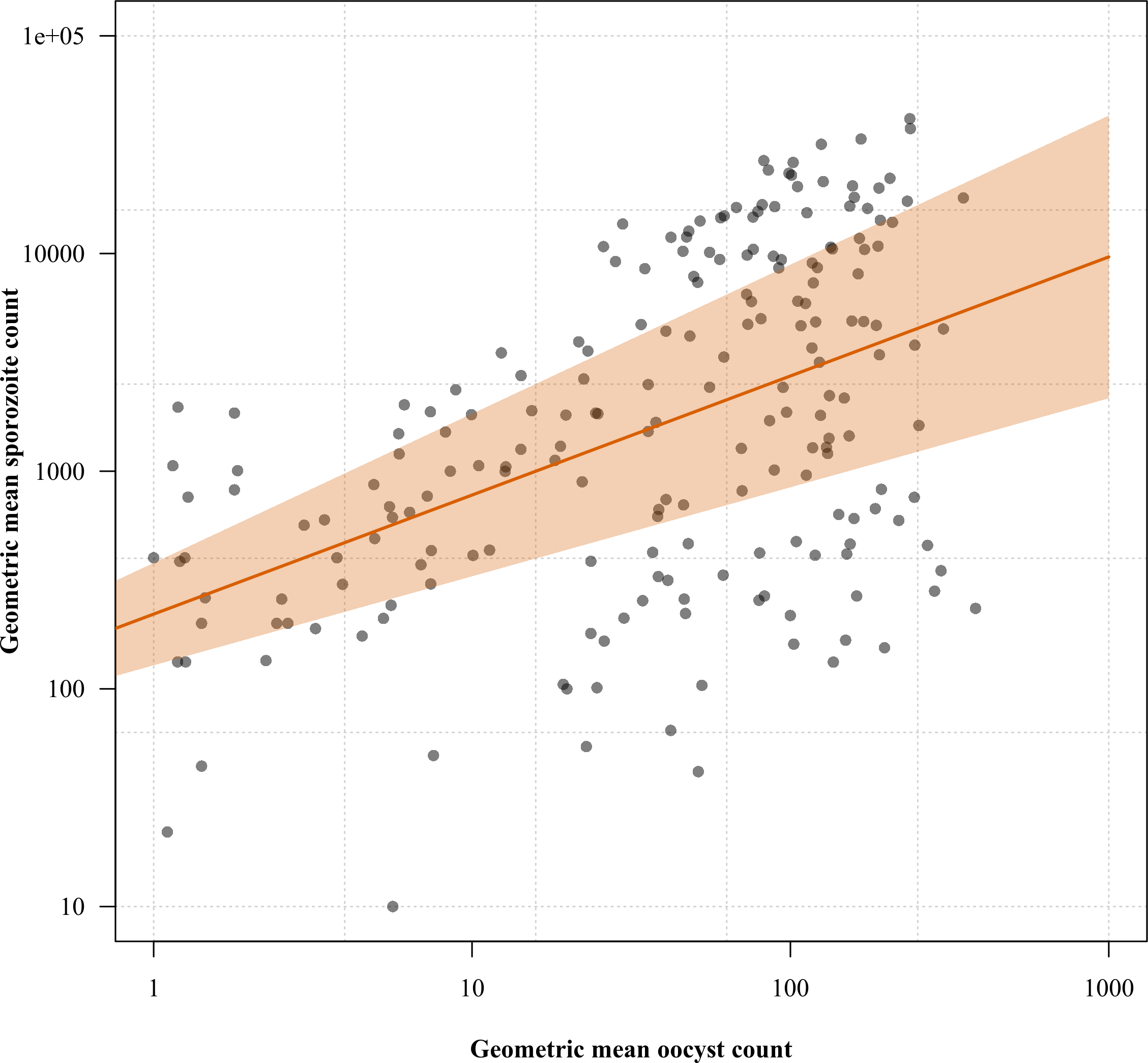
**Figure S2.**

**Relationship between the mean oocyst and sporozoite counts in the assay.** Points show the observed non-negative values plotted on the log scale; the orange line and shaded area show the linear regression fit and corresponding 95% confidence interval, respectively.


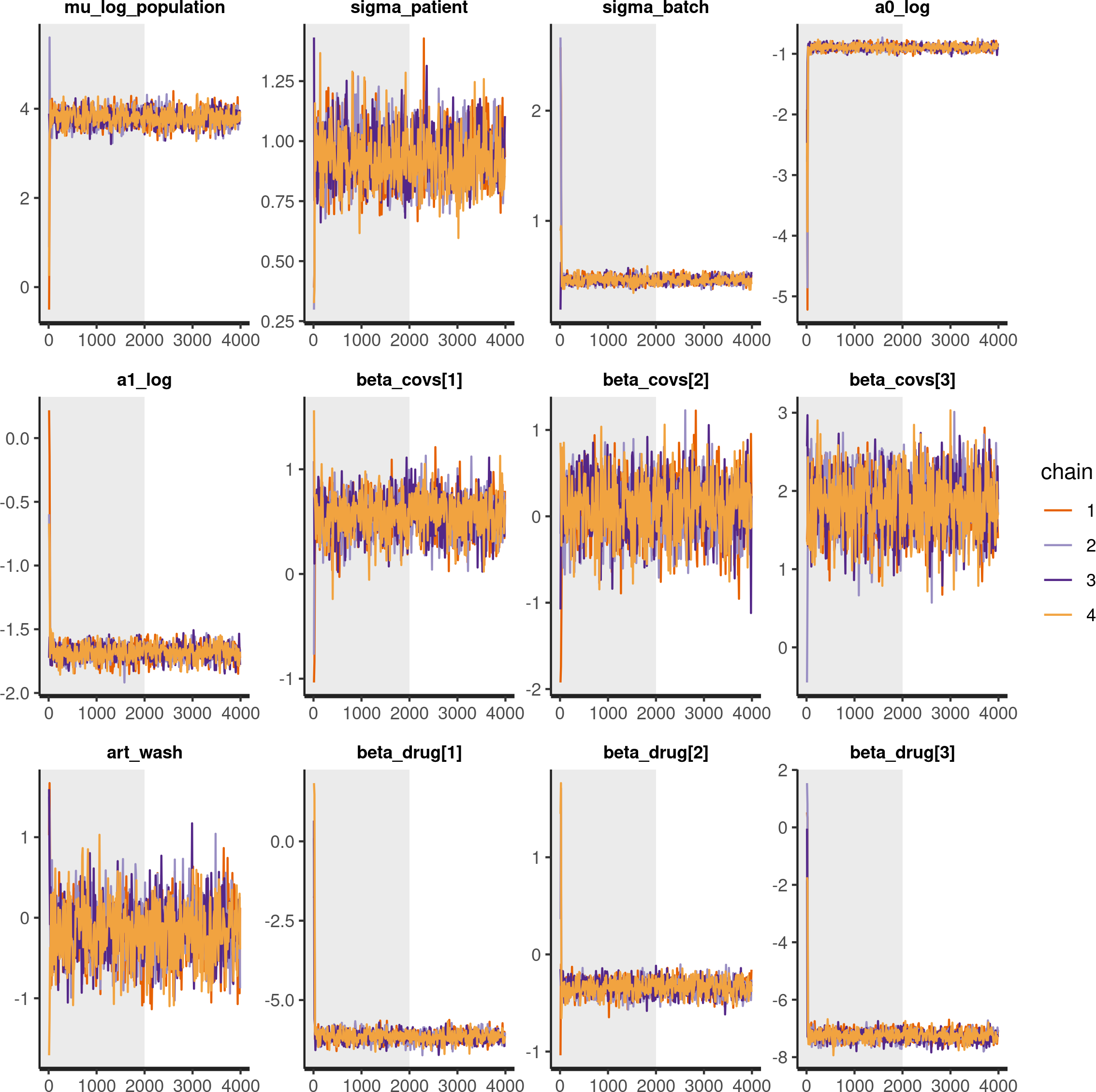
**Figure S3.**

**Traceplot of MCMC chains showing mixing and convergence for the Bayesian multi-level model fitted to oocyst data.**


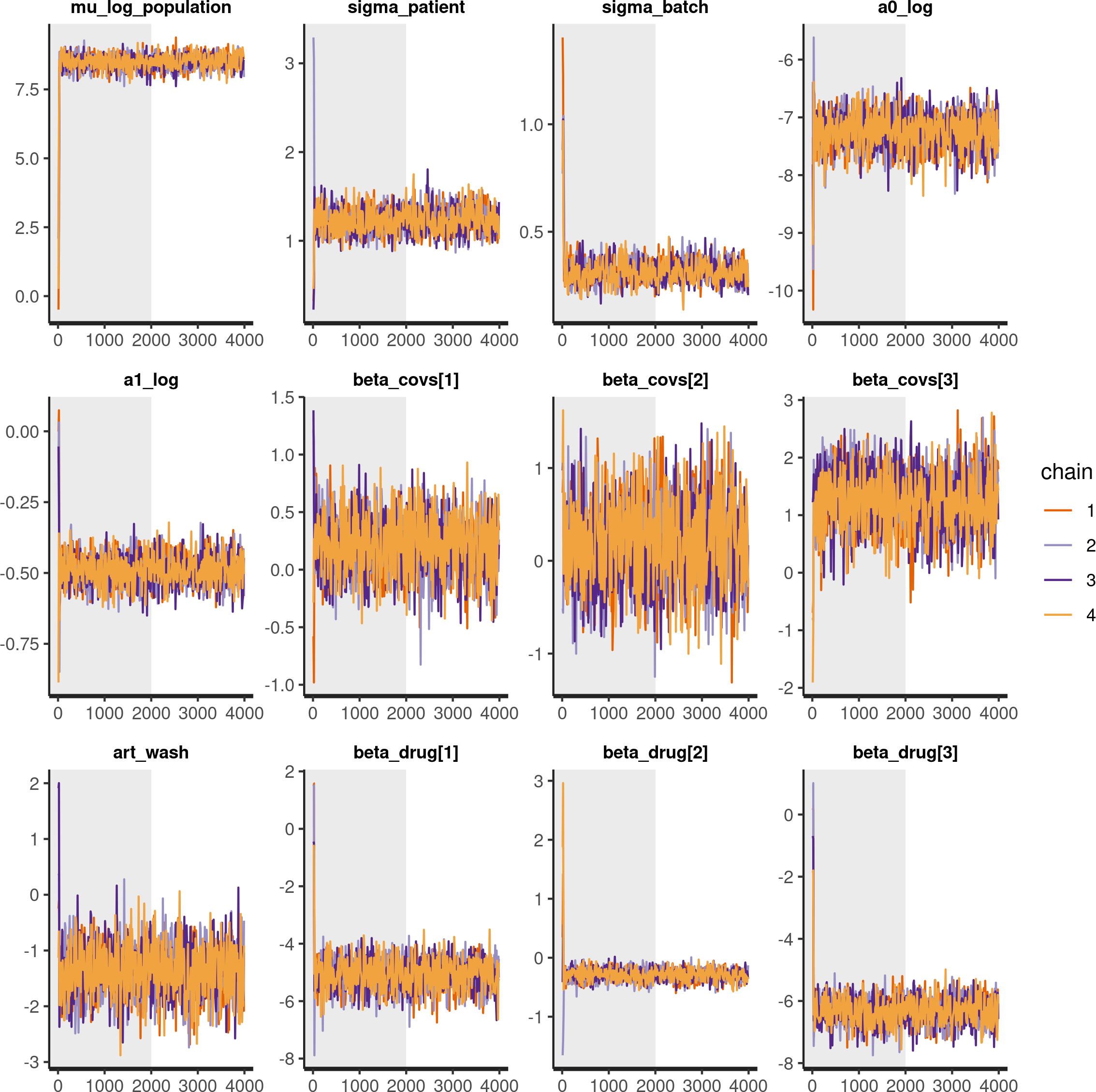
**Figure S4.**

**Traceplot of MCMC chains showing mixing and convergence for the Bayesian multi-level model fitted to sporozoite data.**


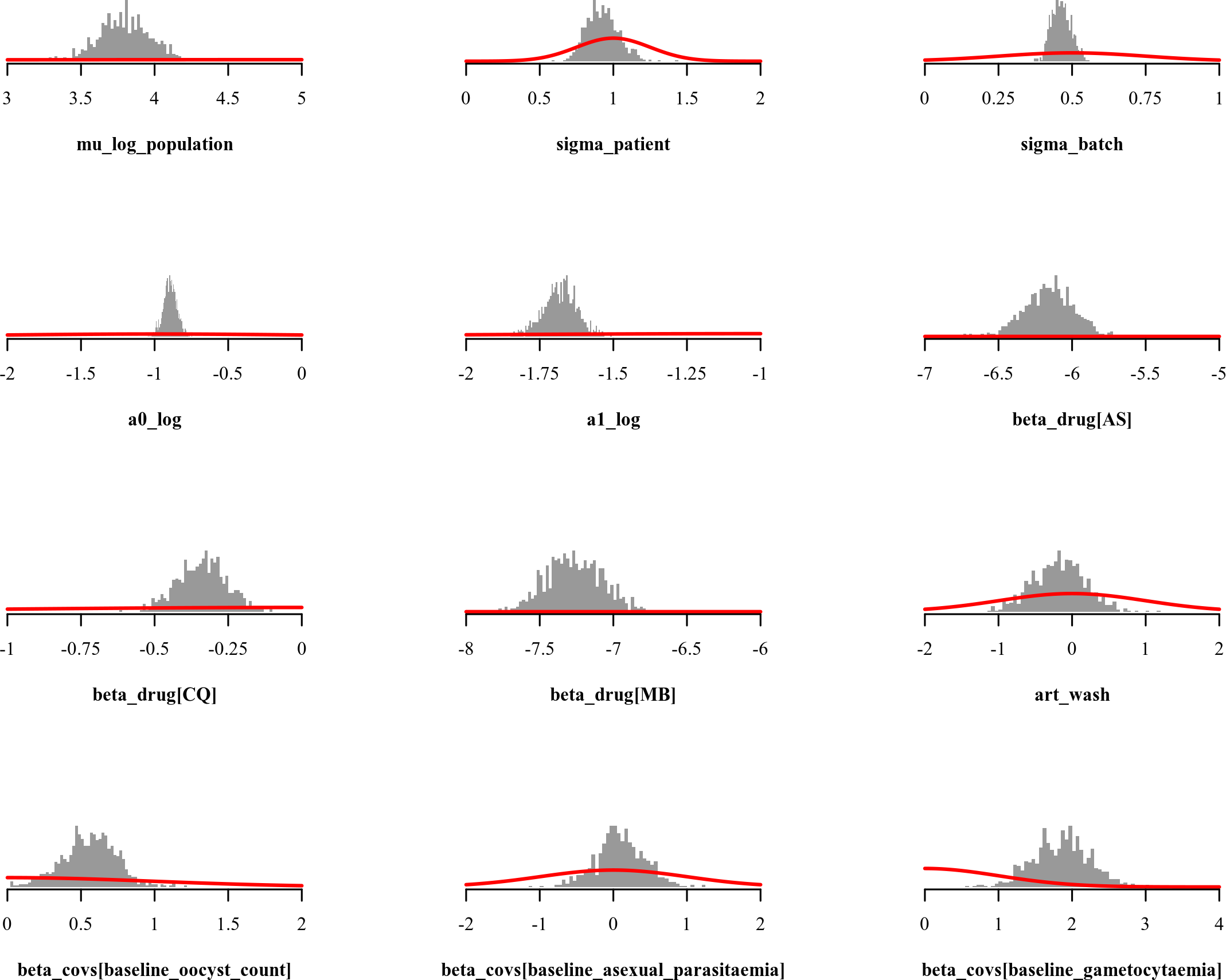
**Figure S5.**

**Comparison between prior distributions (thick red lines) and posterior distributions (shown as histograms) for the parameters of the Bayesian multi-level model fitted to oocyst data.**


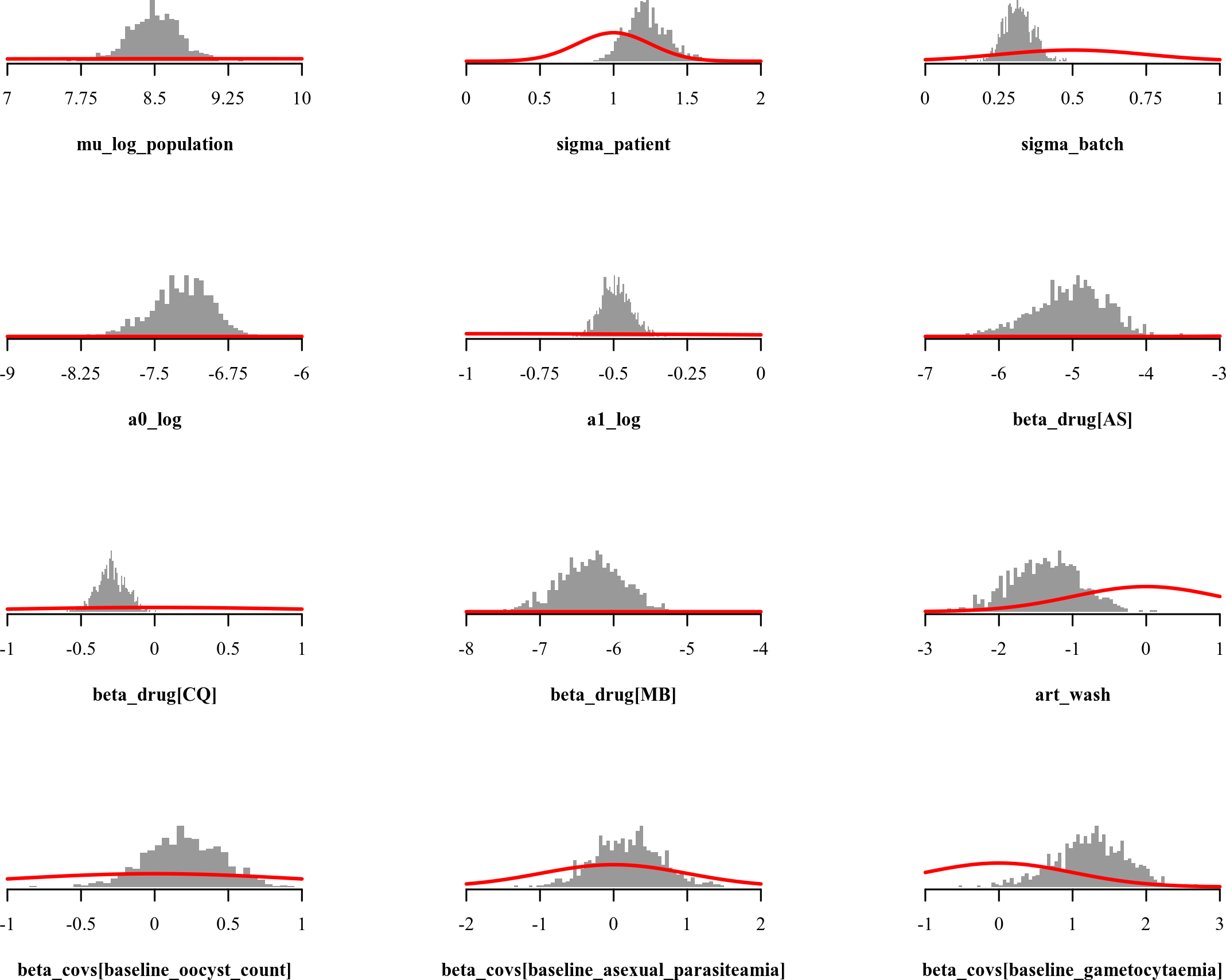
**Figure S6.**

**Comparison between prior distributions (thick red lines) and posterior distributions (shown as histograms) for the parameters of the Bayesian multi-level model fitted to the sporozoite data.**
